# High Seroprevalence of SARS-CoV-2 (COVID-19)-Specific Antibodies among Healthcare Workers: A Cross-Sectional Study in Guilan, Iran

**DOI:** 10.1155/2021/9081491

**Published:** 2021-10-22

**Authors:** Heydar Ali Balou, Tofigh Yaghubi Kalurazi, Farahnaz Joukar, Soheil Hassanipour, Mohammad Shenagari, Mahmoud Khoshsorour, Fariborz Mansour-Ghanaei

**Affiliations:** ^1^Gastrointestinal and Liver Diseases Research Center, Guilan University of Medical Sciences, Rasht, Iran; ^2^Department of Health, Nutrition & Infectious Diseases, School of Medicine, Guilan University of Medical Sciences, Rasht, Iran; ^3^GI Cancer Screening and Prevention Research Center, Guilan University of Medical Sciences, Rasht, Iran; ^4^Caspian Digestive Diseases Research Center, Guilan University of Medical Sciences, Rasht, Iran

## Abstract

**Background:**

This study was conducted to evaluate the anti‐SARS‐CoV‐2 IgM and IgG antibodies among healthcare workers in Guilan.

**Methods:**

This cross-sectional study was conducted on 503 healthcare workers. Between April and May 2020, blood samples were collected from the healthcare workers of Razi Hospital in Rasht, Guilan, Iran. Enzyme-linked immunosorbent assay (ELISA) was used for the detection and quantitation of anti‐SARS‐CoV‐2 IgM/IgG antibodies by using kits made by Pishtaz Teb Company, Tehran, Iran.

**Results:**

From a total of 503 participants, the result of the anti‐SARS‐CoV‐2 IgM antibody test was positive in 28 subjects (5.6%) and the anti‐SARS‐CoV‐2 IgG antibody test was positive in171 subjects (34%). Participants in the age group of 35–54 years were significantly more likely to have a positive anti‐SARS‐CoV‐2 antibody test than the age group of 20–34 years (odds ratio = 1.53, 95% CI: 1.04–2.25, *P*=0.029). Also, physicians were significantly more likely to have a positive antibody test than office workers (odds ratio = 1.92, 95% CI: 1.04–3.54, *P*=0.037). The wide range of symptoms was significantly associated with the positive anti‐SARS‐CoV‐2 antibody test. The most significant association was observed between fever and a positive anti‐SARS‐CoV‐2 antibody test (odds ratio = 3.03, 95% CI: 2.06–4.44, *P* < 0.001).

**Conclusion:**

The results of the current study indicated that the seroprevalence of COVID-19 was high among healthcare workers of Guilan Province. It seems that this finding was due to the earlier exposure to COVID-19 and the lack of awareness and preparedness to deal with the pandemic in Iran, compared to other countries.

## 1. Introduction

In December 2019, an unknown manifestation of pneumonia was detected in Wuhan, China, by a new virus called coronavirus disease 2019 (COVID-19) [1–3]. Compared to SARS in 2002/2003 and MERS virus in 2012/2014, this virus spreads rapidly [4, 5].

The COVID-19 patient had respiratory and sometimes gastrointestinal symptoms, including fever, cough, shortness of breath, muscular pain, dizziness, headache, sore throat, runny nose, chest pain, and diarrhea [6–8]. The main route of transmission was direct and indirect contact with respiratory droplets [9–11].

The diagnostic method for coronavirus infection, including complete blood count with white blood cell differential, CRP, real-time PCR, and chest radiography, had an important role in the evaluation, diagnosis, and treatment of COVID-19 [12–14]. The real-time reverse transcription polymerase chain reaction (RT‐PCR) method could be used to detect COVID-19 from the nasopharyngeal and oropharyngeal swab [15]. The coronavirus RNA test was declared as the standard diagnostic test [16]. However, cases of false negatives have been reported, including 2 cases of failure of this test in quarantined patients, and this can cause a serious return of the virus in the infection transmission cycle [17]. Also, the study by *Pan* et al. revealed that although the RT-PCR test has been proposed as a standard diagnostic method of COVID-19, it has many limitations [18]. In addition, many false negatives were reported from this diagnostic test [18]. Therefore, there is a need for an accurate and rapid testing method, in order to detect the infected patients and asymptomatic carriers rapidly and to prevent the transmission of the disease and ensure timely treatment of patients.

Some studies reported the most common diagnostic methods of COVID-19 in microbiology laboratories were to detect and extract antibodies from serum samples using ELISA [19–22]. In some studies, the IgG immunoglobulin test was used to determine the seroprevalence of SARS infection [23]. Since it is difficult to achieve a reliable assessment of symptomatic patients with the current diagnostic methods, the rapid and accurate diagnostic methods for COVID-19 are needed. Serological analysis is an accurate and efficient method for screening many pathogens, especially specific IgM and IgG antibodies that are detected by ELISA [24, 25].

Measurement of IgG, IgM, and IgA antibody titers can play an important role in diagnosing the acute and chronic stages of the disease. In this regard, a study by Demay et al. demonstrated that detecting anti‐SARS‐CoV‐2 IgM and IgG antibodies was a rapid and easy method for screening COVID-19 [26].

Antibody detection as an effective adjunct to RNA analysis was an important tool for understanding the occurrence, progression, prognosis, and outcome of COVID-19 and had an epidemiological importance [27]. According to a study by Shu et al., IgG antibodies were present in the blood 3 to 40 days after the onset of symptoms [28]. After exposure to pathogens such as viruses, the humoral immune system produces specific antibodies to destroy the pathogens and prevent their progression. In fact, when the virus enters the body, innate immune system cells and factors identify it and immediately inform the adaptive immune system of the presence of this uninvited guest [29]. Innate immune cells, such as macrophages, pick up viral agents and, after processing, deliver them to specific lymphocytes located in the lymph nodes and spleen [30]. First, the IgM antibodies are produced; then, as the immune response progresses and the appropriate signals are received, B lymphocytes become plasma cells which will mostly produce IgG or IgA antibodies [31].

Studies have shown that, in early immune responses, IgM antibody production is predominant with low quantity and short time. In contrast, IgG production is delayed, but its production is higher and will remain in the serum for a longer period of time and remain in the blood even after the infection is gone, due to immune memory [32]. Therefore, the detection of IgM in a suspect's serum could be an immunological evidence of a recent infection. However, the detection of IgG in the serum of an asymptomatic person often indicates a previous infection. In addition, IgM usually begins to decline if the patient is successfully treated [33]. Therefore, a similar algorithm must be observed for COVID-19.

COVID-19 poses an important occupational health risk to healthcare workers, which has attracted global scrutiny [34–36]. As COVID-19 is widely spreading, a rapid and accurate diagnostic method is needed. Serological testing is an accurate and efficient method for screening many pathogens and differentiating between acute and chronic stages of infection. Given that more medical research on the expression of anti-SARS-CoV-2 antibodies and the prognosis of COVID-19 is needed, the aim of this study was to detect the anti‐SARS‐CoV‐2 IgM and IgG antibodies among healthcare workers in Razi Hospital of Guilan, in 2020.

## 2. Methods and Study Design

This cross-sectional study was conducted on 503 healthcare workers of Razi Hospital in Rasht, Guilan (Northern Province of Iran), between April and May 2020.

Razi Hospital, located on Razi Street in Rasht (the center of Guilan Province), is an educational and medical hospital affiliated to Guilan University of Medical Sciences with 204 fixed beds, which was established in 1953. This hospital was selected as the referral center for COVID-19 patients during the peak of the epidemic.

The total number of Razi Hospital healthcare workers was 816, of which more than 60% (503) were randomly entered into the study. The sample size was estimated based on the main outcome, namely, the seroprevalence of SARS-CoV-2-specific antibodies, with a confidence level of 95%.

### 2.1. Ethical Consideration

Informed consent was obtained from each participant. The study protocol was approved by the Ethics Committee of Guilan University of Medical Sciences (Ethics Code: IR. GUMS. REC. 1399.032).

### 2.2. Measurements

Relevant information was collected in 3 parts. The first part included demographic characteristics of participants (including age, education, gender, household size and marital status, and blood group), the second part collected data on the job category of participants (including physician, nurse, service worker, lab staff, and office staff), and the third part collected data on participants' experience of COVID-19 symptoms during the pandemic (including fever, cough, sore throat, dyspnea, runny nose, fatigue, myalgia, arthralgia, headache, chest pain, diarrhea, nausea and vomiting, anosmia, and chills).

5 ml blood samples were collected from all participants. Enzyme-linked immunosorbent assay (ELISA) was used for the detection and quantitation of anti‐SARS‐CoV‐2 IgM/IgG antibodies by using kits made by Pishtaz Teb Company (Pishtaz Teb Diagnostics, Tehran, Iran). The cutoff provided by the manufacturer for positive anti-IgM and -IgG antibody was 1.1.

### 2.3. Statistical Analysis

Descriptive analyses were reported as mean and standard deviation for quantitative variables and number and percentage for qualitative variables. The chi-square test was used to investigate the association between qualitative variables. Logistic regression analyses were performed to identify independent factors associated with a positive antibody test. All analyzes were performed by SPSS version 20 software (SPSS Inc., Chicago, IL, USA). A *P* value less than 0.05 was considered significant.

## 3. Results

A total of 503 healthcare workers were included in this study. The mean age of the subjects was 39.27 with a standard deviation of 10 years. The majority of the participants were female (68.4%) and were married (70.6%).

The result of anti‐SARS‐CoV‐2 IgM antibody test was positive in 28 subjects (5.6%), and the anti‐SARS‐CoV‐2 IgG antibody test was positive in168 subjects (33.3%).

The seroprevalence of anti‐SARS‐CoV‐2 IgM and IgG antibodies according to the characteristics of the study population is shown in Table 1.

The comparison of the seroprevalence of anti‐SARS‐CoV‐2 antibodies in the current study with some other studies is presented in Table 2. The seroprevalence of anti‐SARS‐CoV‐2 antibodies among healthcare workers in Rasht was higher than that among healthcare workers in other countries.

**Figure 1:**
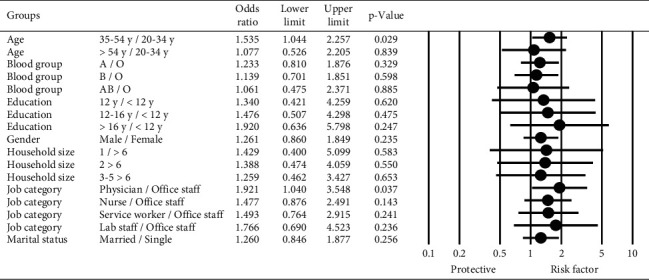
Results of logistic regression to explore factors associated to the seroprevalence of anti‐SARS‐CoV‐2 antibodies.

Figure 1 reveals the results of logistic regression to explore factors associated to the seroprevalence of anti‐SARS‐CoV‐2 antibodies.

Although the result of the anti‐SARS‐CoV‐2 antibody test was 26% more positive in male than female, this finding was not statistically significant (OR = 1.26, 95% CI: 0.86–1.84, *P*=0.235).

Participants in the age group of 35–54 years were significantly more likely to have a positive anti‐SARS‐CoV‐2 antibody test than the age group of 20–34 years (OR = 1.53, 95% CI: 1.04–2.25, *P*=0.029), while this increase was not significant in the age group of more than 54 years than the age group of 20–34 years (OR = 1.07, 95% CI: 0.52–2.20, *P*=0.839).

The comparison of the seroprevalence of anti‐SARS‐CoV‐2 antibodies in job categories showed physicians were significantly more likely to have a positive anti‐SARS‐CoV‐2 antibody test than office workers (OR = 1.92, 95% CI: 1.04–3.54, *P*=0.037) while there was no significant difference between other job categories and office workers.

The positive anti‐SARS‐CoV‐2 antibody test was more common in the A blood group, but this finding was not statistically significant (OR = 1.23, 95% CI: 0.81–1.87, *P*=0.329).

More educated participants reported a higher seroprevalence of anti‐SARS‐CoV‐2 antibodies, but this finding was not statistically significant (OR = 1.92, 95% CI: 0.63–5.79, *P*=0.247).

There was no independent association of household size and marital status with the seroprevalence of anti‐SARS‐CoV‐2 antibodies.

**Figure 2:**
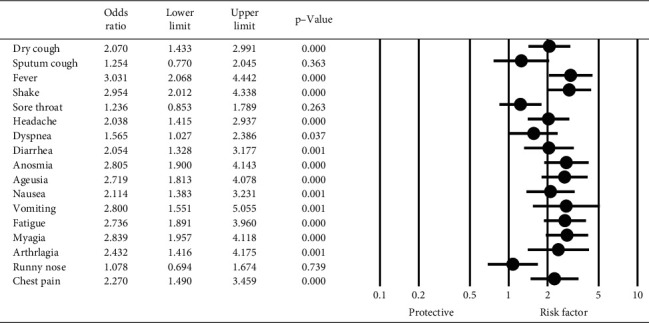
The results of logistic regression to explore the relationship between the symptoms of COVID-19 and the positive anti‐SARS‐CoV‐2 antibody.

The results of logistic regression to explore the relationship between the symptoms of COVID-19 and the positive anti‐SARS‐CoV‐2 antibody test are shown in Figure 2. The wide range of symptoms was significantly associated with the positive anti‐SARS‐CoV‐2 antibody test. The most significant association was observed between fever and a positive anti‐SARS‐CoV‐2 antibody test (OR = 3.03, 95% CI: 2.06–4.44, *P* < 0.001). Only sputum cough, runny nose, and sore throat were not significantly associated with a positive anti‐SARS‐CoV‐2 antibody test.

## 4. Discussion

This study was conducted in one of the referral hospitals for COVID-19 patients during the peak of the epidemic in Guilan Province.

Our study demonstrates that the unweighted seroprevalence of anti‐SARS‐CoV‐2 IgM and IgG antibodies in our study population was 5.6% and 33.3%, respectively, and the unweighted seroprevalence of the combination of both IgM and IgG antibodies was 39%. These findings indicate that the seroprevalence of anti‐SARS‐CoV‐2 antibodies was higher in the healthcare workers compared to the general population of Guilan Province, which was reported to be 22% in Shakiba et al.'s study [37]. Other studies in the general population reported that the seroprevalence of anti‐SARS‐CoV‐2 antibodies was 5% in Spain [38], 2.7% in Milan, Italy [39], 4% in California [40], 7.6% in Daegu, South Korea [41], and 1.7% in Luxembourg [42]. In a similar study of healthcare workers in Spain [43] and Pakistan [44], the seroprevalence was 9.3% and 8.3%, respectively. Signorelli et al. showed that the estimated period-prevalence of COVID-19 in Italy varies from 0.35% in Sicily to 13.3% in Lombardy [45]. In Rostami et al.'s meta-analysis study about SARS-CoV-2 worldwide seroprevalence, results varied from 1.45% in South America to 5.27% in Northern Europe. The findings suggested an association of seroprevalence with human development indices, income levels, and climate [46]. Although the high seroprevalence of anti‐SARS‐CoV‐2 antibodies in our study population may be due to the higher risk of contact in healthcare workers than the general population, conducting the study at the peak of the COVID-19 pandemic (March to April) and the lack of preparedness to deal with the pandemic in Iran and Rasht can be other reasons for this high prevalence.

Our findings revealed that the seroprevalence of anti‐SARS‐CoV‐2 antibody was higher in males than that in females (42.8 vs. 37.2%, respectively), but it was not statistically significant. This finding was in line with a study in the general population of Switzerland (7.3 in male vs. 4.7% in female), a study in the general population of California (5.18 in male vs. 3.31% in female), and a study in the general population of South Korea (11% in male vs. 4% in female) [40, 41, 47]. In some studies in Spanish healthcare workers, positive anti‐SARS‐CoV‐2 antibody tests were more prevalent in females, but it was not significant [43]. In a study in the general population of Guilan, Iran, the seroprevalence of anti‐SARS‐CoV‐2 antibody was 23% in females and 22% in males [37].

In the current study, positive anti‐SARS‐CoV‐2 antibody tests were significantly more prevalent in the age group of 35–54 years. In other studies, the highest seroprevalence of anti‐SARS‐CoV‐2 antibody was in the following age groups: 20–34 years in a Spanish population, 20–49 years in a Swiss population, more than 60 years in a South Korean population, and 35–54 years in a California population [38, 40, 41, 47].

We found that the highest prevalence of a positive anti‐SARS‐CoV‐2 antibody test was in physicians. In the study of Spanish healthcare workers, the highest prevalence was in physicians, followed by nurses and laboratory staff [43]. In another study in Pakistan and Spain, the highest prevalence of a positive anti‐SARS‐CoV‐2 antibody test was in healthcare workers (17% and 10.2%, respectively) compared to other job categories [38, 44]. In a similar study conducted in Guilan, healthcare workers had a higher seroprevalence of anti‐SARS‐CoV‐2 antibody than the general population (29% vs. 22%) [37]. In Deiana et al.'s study, of the 1371 positive cases analyzed, 323 (23.5%) are healthcare workers and 563 (41.1%) reside in social or healthcare facilities [48]. Another study in Saudi Arabia was conducted between May 20 and 30, 2020, showing that the overall positivity rate by the immunoassay was 299 (2.36%) with a significant difference between the control group (0.8%) and case-hospital group (2.9%) (*P* value < 0.001) [49]. Kayı et al. in Belgian public multiple-site hospital revealed that the overall seroprevalence was 7.6% and higher seroprevalence was seen in nurses (10.0%) than in physicians (6.4%), paramedical (6.0%), and administrative staff (2.9%). A review study conducted by Kayı et al. indicates a SARS-CoV-2 seroprevalence rate of 8% among studies that included >1000 HCWs for the year 2020, before vaccinations started [50]. The probable cause for this finding is the higher risk of exposure to the virus in healthcare workers compared to other population groups.

Our study revealed that the highest prevalence of a positive anti‐SARS‐CoV‐2 antibody test was in participants with more than 16 years of education, which can be justified by considering that the physicians and nurses are more exposed to patients than other healthcare workers.

In the current study, we found no association between household size and the seroprevalence of anti‐SARS‐CoV‐2 antibodies. In Spain, the highest prevalence of a positive anti‐SARS‐CoV‐2 antibody test was reported in households with 2 members [38]. In a meta-analysis study, the most common risk factors associated with higher seroprevalence rate in HCWs were ethnicity, male gender, and having a higher number of household contacts [50]. This finding should be interpreted with caution because the house area may be more important than the household size.

Our results revealed that the highest prevalence of a positive anti‐SARS‐CoV‐2 antibody test was observed in participants with blood group A, but this finding was not statistically significant. In similar seroepidemiological studies, blood group was not investigated. However, the results of some studies reported that the highest risk of COVID-19 mortality was in people with blood group A [51], which could be in line with our findings.

According to the results of our study, fever was the most common symptom associated with a positive anti‐SARS‐CoV‐2 antibody test. The study by Sood et al. revealed that fever with an odds ratio of 2.8 and anosmia with an odds ratio of 4.1 were most associated with a positive anti‐SARS‐CoV‐2 antibody test [40]. In the study by Streeck et al., fever (OR = 4.6), dry cough (OR = 2.8), and anosmia (OR = 18.5) were the most prevalent symptoms associated with a positive anti‐SARS‐CoV‐2 antibody test [52]. In the study by Liu et al., the most common symptoms associated with mortality were fever, followed by cough and sputum [53]. The probable cause of these findings might be that symptoms such as dry cough, anosmia, and fever were more specific for COVID-19 than other similar diseases such as cold and flu.

## 5. Conclusions

The results of the current study indicated that the serological prevalence of COVID-19 was high among healthcare workers of Guilan Province. It seems that this finding was due to the earlier exposure to COVID-19 and the lack of awareness and preparedness to deal with the pandemic in Iran, compared to other countries.

## Figures and Tables

**Table 1:** Unweighted characteristics of study participants and proportion with IgM or IgG for COVID-19.

Characteristics	Subgroup	Sample size	Proportion of the sample, % (95% CI)	No. of positive cases	Unweighted proportion positive for IgM or IgG, % (95% CI)	Unweighted proportion positive for only IgM, % (95% CI)	Unweighted proportion positive for only IgG, % (95% CI)
Entire sample	—	503	100	196 (168 IgG, 28 IgM)	39.0 (34.8–43.3)	5.6 (3.8–8.0)	33.3 (29.3–37.7)
Gender	Male	159	31.6	68 (58 IgG, 10 IgM)	42.8 (35.0–50.8)	6.3 (3.2–11.5)	36.4 (29.1–44.5)
	Female	344	68.4	128 (110 IgG, 18 IgM)	37.2 (32.1–42.6)	5.2 (3.2–8.3)	31.9 (27.1–37.2)
Age, y	20–34 y	189	37.6	63 (51 IgG, 12 IgM)	33.3 (26.7–40.6)	6.3 (3.4–11.0)	27.0 (20.9–34.0)
	35–54 y	274	54.5	119 (104 IgG, 15 IgM)	43.4 (37.5–49.5)	5.5 (3.2–9.0)	37.9 (32.2–44.0)
	>54 y	40	8.0	14 (13 IgG, 1 IgM)	35.0 (21.1–51.7)	2.5 (0.1–14.7)	32.5 (19.0–42.9)
Education	<12 y	17	3.4	5 (4 IgG, 1 IgM)	29.4 (11.3–55.9)	5.9 (3.1–30.7)	29.4 (11.3–55.9)
	12 y	67	13.3	24 (22 IgG, 2 IgM)	35.8 (24.7–48.5)	3.0 (0.1–11.3)	32.8 (22.1–45.5)
	12–16 y	302	60.0	115 (94 IgG, 21 IgM)	38.1 (32.6–43.8)	7.0 (4.4–10.6)	31.1 (26.0–36.7)
	>16 y	117	23.3	52 (46 IgG, 6 IgM)	44.4 (35.3–53.9)	5.1 (2.1–11.2)	39.3 (30.0–48.8)
Blood group	A	171	34.0	71 (60 IgG, 11 IgM)	41.5 (34.1–49.3)	6.4 (3.4–11.5)	35.1 (28.1–42.8)
	B	106	5.8	42 (38 IgG, 4 IgM)	39.6 (30.4–49.6)	3.8 (0.1–9.9)	35.8 (26.9–45.8)
	AB	29	21.1	11 (10 IgG, 1 IgM)	37.9 (21.3–57.6)	3.4 (0.1–19.6)	34.5 (18.6–54.3)
	O	197	39.2	72 (60 IgG, 12 IgM)	36.5 (29.9–43.7)	6.1 (3.3–10.6)	30.4 (24.2–37.4)
Marital status	Single	148	29.4	52 (47 IgG, 5 IgM)	35.1 (27.6–43.4)	3.4 (1.2–8.1)	31.7 (24.5–40)
	Married	355	70.6	144 (121 IgG, 23 IgM)	40.6 (35.4–45.9)	6.5 (4.2–9.7)	34.1 (29.2–39.3)
Job category	Physician	90	17.9	41 (35 IgG, 6 IgM)	45.6 (35.1–56.3)	6.7 (2.7–14.5)	38.9 (31.0–52.0)
	Nurse	235	46.7	92 (76 IgG, 16 IgM)	39.1 (32.9–45.7)	6.8 (4.0–11.0)	32.3 (27.7–40.1)
	Service worker	66	13.1	26 (24 IgG, 2 IgM)	39.4 (27.8–52.2)	3.0 (0.1–11.5)	36.3 (25.1–49.1)
	Lab staff	23	4.6	10 (8 IgG, 2 IgM)	43.5 (23.9–65.1)	8.7 (0.2–29.5)	34.7 (17.2–57.1)
	Office staff	89	17.7	27 (25 IgG, 2 IgM)	30.3 (21.2–41.1)	2.2 (0.3–8.6)	28.0 (19.3–38.7)
Household size, residents	1	24	4.8	10 (9 IgG, 1 IgM)	41.7 (22.8–63.0)	4.2 (0.2–22.1)	37.5 (19.5–59.2)
	2	83	16.5	34 (30 IgG, 4 IgM)	41.0 (30.4–52.3)	4.8 (1.5–12.5)	36.1 (26.1–47.5)
	3–5	378	75.1	146 (124 IgG, 22 IgM)	38.6 (33.7–43.7)	5.8 (3.7–8.8)	32.8 (28.1–37.8)
	≥6	18	3.6	6 (5 IgG, 1 IgM)	33.3 (14.3–58.8)	5.6 (0.1–29.3)	27.8 (10.7–53.6)

**Table 2:** Comparison of the results of other studies with the current research.

Author, year	Country	Population	Sample size	IgG	IgM	IgG or IgM	Date of sampling	The onset of the epidemic
Pollán, 2020	Spain	Healthcare	1109	10.2%	—	—	April 27 to May 11, 2020	March 2020
Javed, 2020	Pakistan	Healthcare	3120	4.6%	4.1%	8.3%	NM	April 2020
Valenti, 2020	Italy (Milan)	Healthcare	37	—	—	5.4%	February 24 to April 8, 2020	March 2020
Comar, 2020	Italy (Trieste)	Healthcare	727	—	—	17.2%	April 2020	March 2020
Garcia-basteiro, 2020	Spain (Barcelona)	Healthcare	578	—	—	9.3 and	From 28 March to 9 April 2020	March 2020
Alserehi, 2020	Saudi Arabia	Healthcare	12621			2.3%	May 2020	March 2020
Venugopal, 2020	United States (New York City)	Health care	500			27%	May 2020	April 2020
Amendola, 2020	Italy (Lombardy)	Healthcare	742	5.13%			April 2020	March 2020
Yogo, 2020	United States (San Diego)	Healthcare	1770			2.2%	May 2020	March 2020
Shakiba, 2020	Iran (Guilan)	Healthcare	44	—	—	29%	April 2020	February 2020
Our study	Iran (Guilan)	Healthcare	503	33.3%	5.6%	39%	April 2020	February 2020

## Data Availability

The datasets analyzed during the current study are available from the corresponding author on reasonable request.

## Abbreviations

HCW: Healthcare workers
ELISA: Enzyme-linked immunosorbent assay
COVID-19: Coronavirus disease 2019.
